# Supplementary material for: Strain-Specific Therapeutic Potential of Lactiplantibacillus plantarum: A Systematic Scoping Review
Source: Nutrients. 2025 Mar 27;17(7):1165. doi: 10.3390/nu17071165 (PMC11990516; doi:10.3390/nu17071165)
Supplement: Supplementary file 1 [file nutrients-17-01165-s001.zip › nutrients-3511507-supplementary.pdf]

Item	Lactobacillus plantarum Strains	Authors (Published Year)	Study Type (Single blind, Double blind, etc.)	Website	Country	Samples Size (Details : Size, Sex, Age, etc.)	Group Study Allocation (No. of groups, Size of each group, Ratio of Intervention : Control (I : C))	The Diseases of The Study	Duration of the Intervention
1	ECGC 13110402	Adele Costabile (2017)	Conducted randomized, double-blind, placebo-controlled trial	https://doi.org/10.1371/journal.pone.0187964	United Kingdom	49 normal to mildly hypercholesterolaemic adults, potential healthy volunteers (aged from 18 to 50 years)	The study was carried out in a single-centre, n=46, who completed the study. Randomly assigned into 2 groups of placebo and active groups. (I : C = 23 : 23)	Investigated the cholesterol reducing capacity of Lactobacillus plantarum ECGC 13110402	Treatment period for 12 weeks.
2	299v	Agnieszka Olek (2017)	A prospective, double-blind, randomized, placebo-controlled, multicenter, parallel-group study	https://doi.org/10.1016/j.jpeds.2017.03.047	Poland	A total of 438 children were randomized to receive LP299V (n= 218) or the placebo (n = 220).	I : C = 218 : 220	LP299V did not show a significant effect on the secondary outcomes related to abdominal symptoms (pain, vomiting, flatulence, and distension) compared with placebo.	The total duration of the study varied from 15 to 28 days.
3	IS-10506	Alpha Fardah Athiyyah (2019)	A randomized, double-blind, placebo-controlled study was conducted	http://www.ncbi.nlm.nih.gov/pmc/articles/pmc6635306/	Indonesia	A total of 21 subjects were randomly assigned to intervention (n=10) and the placebo group (n=11).	I : C = 10 : 11	IS-10506 reduced the blood LPS level but showed no effect on the humoral mucosa and systemic immune response in HIV-infected children undergoing ARV therapy.	6 weeks intervention period. It was conducted from December 2012 to March 2013.
4	299v	Anna Berggren (2003)	The study was parallel, double-blind.	http://dx.doi.org/10.1080/08910600410024825	Sweden	69 children were randomly assigned to intervention (n=33) and the placebo (n=36)	I : C = 33 : 36	The children tolerated the fermented oat product well, the faecal microflora was positively altered and the children's nutritional intake was improved.	The first 2 weeks were a baseline period where no product was consumed, followed by 3 weeks test period
5	299v	Bengt Klarin (2008)	A randomised controlled open pilot study	https://doi.org/10.1186/cc7109	Sweden	The 44 critically ill patients on mechanical ventilation were randomised to either intervention (n=23) or the placebo (n=21)	I : C = 10 : 10	It found no difference between the effect of Lp299 and CHX (control) used in oral care procedures,	Sampling was repeated on days 2, 3, 5, 7, 10, 14 and 21.

Item	Lactobacillus plantarum Strains	Authors (Published Year)	Study Type (Single blind, Double blind, etc.)	Website	Country	Samples Size (Details : Size, Sex, Age, etc.)	Group Study Allocation (No. of groups, Size of each group, Ratio of Intervention : Control (I : C))	The Diseases of The Study	Duration of the Intervention
6	CCFM1143	Bo Yang, Yue Yue (2021)	Conducted randomized, double-blind, placebo-controlled trial	https://doi.org/10.3389/fimmu.2021.746585	China	55 patients with chronic diarrhea were randomly assigned into the probiotic group (n = 28) and the placebo group (n = 27) for treatment in 4 weeks, respectively.	I : C = 28 : 27	This probiotics showed clinical effectiveness in managing chronic diarrhea	The Experimental preparation period was 1 week and the experimental process was 4 weeks, with a total duration of 5 weeks.
7	CJLP243 (KCCM11045P)	Byung Jun Yoon (2021)	Conducted randomized, double-blind, placebo-controlled trial	https://doi.org/10.1111/codi.15463	Korea	36 patients (probiotics, n = 17; placebo, n = 19) completed the primary outcomes.	I: C = 17:19	The probiotic showed tendencies toward improving bowel function and quality of life.	Between June 2016 and March 2018, a total of 36 patients completed the trial. The intervention included 3 weeks.
8	IS-10506	C. R. S. Prakoeswa (2022)	A randomized double-blind placebo-controlled trial study.	https://doi.org/10.1080/09546634.2020.1836310	Indonesia	The 30 adults with mild and moderate atopic dermatitis (AD) were randomly assigned to intervention (n=15) and the placebo (n=15).	I : C = 15 : 15	The administration of LP IS-10506 is effective for alleviating atopic dermatitis (AD) symptoms in adults owing to its immunomodulatory effects.	The 8 weeks intervention period.
9	IS-10506	C.R.S. Prakoeswa (2017)	A randomised double-blind placebo controlled trial	https://doi.org/10.3920/bm2017.0011	Indonesia	22 Atopic dermatitis (AD) children were randomly divided into intervention and control groups of n=12 and n=10 patients, respectively.	I : C = 12 : 10	IS-10506 offered a potential treatment for children with Atopic dermatitis (AD).	12 weeks intervention period.
10	PS128	Chang-Chun Wu (2021)	A randomized clinical trial with a double-blind, placebo-controlled.	https://doi.org/10.3390/nu13113698	Taiwan	A total of 57 subjects were randomly assigned to intervention (n=28) and the placebo intervention (n=29).	I : C = 28 : 29	The probiotics may not have tic-reducing effects in children with Tourette syndrome, but may improve attention deficit and hyperactivity disorder (ADHD).	The 2 months intervention period. The study from 1 August 2017 through to 31 January 2019

Item	Lactobacillus plantarum Strains	Authors (Published Year)	Study Type (Single blind, Double blind, etc.)	Website	Country	Samples Size (Details : Size, Sex, Age, etc.)	Group Study Allocation (No. of groups, Size of each group, Ratio of Intervention : Control (I : C))	The Diseases of The Study	Duration of the Intervention
11	Lp3a	Changliang Zhang (2022)	Randomized, placebo-controlled, double-blind trial.	https://doi.org/10.21037/atm-22-458	China	120 patients with Functional constipation (FC) were randomized to treatment group or control groups (n=60 each)	I: C = 60:60	L. plantarum Lp3a, led to clinically significant improvements in Functional Constipation (FC)	7 days treatment period from July 5 to August 16, 2019.
12	Lpla33 (DSM34428)	Christopher J Martoni (2023)	A randomized, double-blind, placebo-controlled, multi-center, and parallel-arm study, including two doses of the probiotic product and one placebo arm.	https://doi.org/10.3748/wjg.v29.i28.4451	Denmark	Three hundred and seven adults, 18-70 years of age, with IBS-D, were allocated for placebo (n=104), for Lpla33 1B (n=104), and for Lpla33 10B (n=99)	I (1B) : I (10B) : C = 104 : 99 : 104	Lpla33 (DSM34428) is well tolerated and improves IBS symptom	The 8 weeks intervention period
13	LRCC5310	Do Young Shin (2020)	A randomized, placebo-controlled study.	https://doi.org/10.1097/md.0000000000022192	Korea	The enrolled patients were randomly grouped for probiotic group (Group I, n=15), for control group (Group II, n=8). Additionally, there is another group for the medical records of patients (Group III, n=27).	For the enrolled patients, I : C = 15 : 8.	LRCC5310 improved clinical symptoms, including diarrhea and Vesikari score, and inhibited viral proliferation in rotaviral gastroenteritis.	The study period from January 2018 to April 2019. The 7 days study period.
14	HY7714	Dong Eun Lee (2015).	A randomized, double-blind, and placebo-controlled trial	https://doi.org/10.4014/jmb.1509.09021	Republic of Korea	110 volunteers were randomly assigned to intervention n=61) and the placebo group (n=49).	I : C = 61 : 49	The probiotics helps improve the skin elasticity, therefore it confirms of the anti-aging benefit to the skin as a nutricosmetic agent.	12 weeks intervention period.

Item	Lactobacillus plantarum Strains	Authors (Published Year)	Study Type (Single blind, Double blind, etc.)	Website	Country	Samples Size (Details : Size, Sex, Age, etc.)	Group Study Allocation (No. of groups, Size of each group, Ratio of Intervention : Control (I : C))	The Diseases of The Study	Duration of the Intervention
15	299v	Edward Litton (2021).	The study was a parallel group, placebo-controlled, randomised clinical trial.	https://doi.org/10.1007/s00134-020-06322-w	Australia	Total of 221 subjects were randomly assigned to intervention (n=113) and the placebo (n=108).	I : C = 113 : 108	The early and sustained administration of probiotic therapy with 299v to adult patients admitted to the ICU did not result in a significant difference in days alive and at home to Day 60.	The 60 days intervention period.
16	Dad-13	Endang Sutriswati Rahayu (2021)	A randomized, double-blind, placebo-controlled study.	https://doi.org/10.3748/wjg.v27.i1.107	Indonesia	Sixty overweight volunteers were randomly assigned to intervention and placebo group (n= 30 each).	I : C = 30 : 30	A significant decrease in body weight and BMI (P < 0.05) was determined in the treatment group. Dad-13 also caused the Firmicutes population to decrease and the Bacteroidetes population (especially Prevotella) to increase.	90 days intervention period.
17	P17630	F De Seta (2014)	A retrospective comparative study.	https://doi.org/10.1016/j.ejogrb.2014.09.018	Italy	89 women with a diagnosis of vulvovaginal candidiasis (VVC), who were placed into two groups, intervention and control, on the basis of reported treatment.	N.A.	The study confirm that this specific strain as a potential empirical preventive agent for reducing vaginal discomfort after conventional treatment of acute vulvovaginal candidiasis (VVC).	The study period was between January and November 2013.
18	HEAL9	Gunilla Önning (2020)	A randomized, double blind, placebo-controlled study	https://doi.org/10.1016/j.physbeh.2020.113083	Sweden	63 participants completed the study (LPHEAL9, n = 32; placebo, n = 31)	I : C = 32 : 31	intake of LPHEAL9 for four weeks may reduce inflammatory markers coupled to acute stress in chronically stressed individuals.	The study duration was 6 weeks divided in two periods: 1) A run-in period of two weeks and 2) An intervention period of 4 weeks. The study was performed between September 2017 and April 2018

Item	Lactobacillus plantarum Strains	Authors (Published Year)	Study Type (Single blind, Double blind, etc.)	Website	Country	Samples Size (Details : Size, Sex, Age, etc.)	Group Study Allocation (No. of groups, Size of each group, Ratio of Intervention : Control (I : C))	The Diseases of The Study	Duration of the Intervention
19	HEAL9	Gunilla Önning (2023)	A randomized, double-blinded, placebo-controlled, and parallel-designed study	https://doi.org/10.3390/nu15153466	Sweden	One hundred and twenty-nine subjects were randomized to intervention (n = 65) or placebo (n = 64) for 12 weeks.	I : C = 65 : 64	Intake of LPHEAL9 significantly improved cognitive functions compared to the placebo, potentially by ameliorating aspects of mood and sleep.	The 12 weeks intervention period. The study period between June 2021 and March 2022.
20	DR7	Guoxia Liu (2020)	A randomized, double-blind, and placebo-controlled study	https://doi.org/10.3390/ijms21134608	China	Total of 124 subjects, allocated to the placebo n=62 and allocated to DR7 intervention for n=62.	I : C = 62 : 62	Modulated Bowel Movement and Gut Microbiota Associated with Dopamine and Serotonin Pathways in Stressed Adults	12 weeks study period.
21	DR7	H.X. Chong (2018)	A randomised, double-blind, placebo-controlled study	https://doi.org/10.3920/bm2018.0135	Malaysia	One hundred and eleven subjects were randomly assigned to intervention (n=56) and the placebo (n=55).	I : C = 56 : 55	DR7 (in stressed adults) have resulted in reduced symptoms of stress and anxiety, accompanied by improvement of several cognitive and memory functions, and reduced levels of plasma cortisol.	12 weeks intervention period.
22	LB244R®	Helena Falholt Elvebakken (2023)	The trial was designed as a single-center study of a topical ointment.	https://doi.org/10.1111/jocd.15657	Denmark	In total, 23 subjects were included for the study.	No Placebo group. I = 23 subjects included.	It demonstrating the potential of the probiotic ointment (PO) as an anti-aging agent.	The intervention for 56 days. The study was conducted from the 1st of June 2022 to the 1st of August 2022.

Item	Lactobacillus plantarum Strains	Authors (Published Year)	Study Type (Single blind, Double blind, etc.)	Website	Country	Samples Size (Details : Size, Sex, Age, etc.)	Group Study Allocation (No. of groups, Size of each group, Ratio of Intervention : Control (I : C))	The Diseases of The Study	Duration of the Intervention
23	PS128	Hui-Mei Chen (2021)	This study was an open trial with small sample size.	https://doi.org/10.3390/nu13113731	Taiwan	11 patients with major depression disorder (MDD) and received PS128 for intervention.	N.A.	The study found that depressive severity in patients with major depression disorder (MDD) significantly ameliorated, but markers of inflammation, gut permeability, and the composition of gut microbiota did not significantly change.	8 weeks intervention period.
24	DR7	Hui-Xian Chong (2019)	A randomized, double-blind, and placebo-controlled human study	https://doi.org/10.3168/jds.2018-16103	Malaysia	A total of 109 subjects completed the study (Intervention n=56, placebo n=53).	I : C = 56 : 53	The DR7 alleviated the symptoms of upper respiratory tract infections (URTI)	12 weeks study period.
25	CJLP133	J. Kim, B.S. Lee (2017)	N.A.	https://doi.org/10.3920/bm2017.0034	Republic of Korea	76 children were completed the study, all patients were given Lactobacillus plantarum CJLP133.	All patients were given Lactobacillus plantarum CJLP133.	The patients with a specific atopic dermatitis (AD) phenotype showing an immunologically active state may benefit from CJLP133.	12 weeks intervention period.
26	Inducia	J. Štšepetova (2023)	In two parallel-armed, double-blind and placebo-controlled intervention.	https://doi.org/10.3920/bm2022.0030	Estonia	For Jog 4BC intervention, the Inducia group n=82, the placebo, n= 71. For Jog 5 intervention, the Inducia group n=67, the placebo, n= 65	For Jog 4BC intervention, I : C = 82 : 71. For Jog 5 intervention, I : C = 67 : 65.	Inducia expresses antioxidative effect on blood lipids, also demonstrating an anti-cholesterolemic impact and anti-glycaemic profile.	The 8 weeks intervention

Item	Lactobacillus plantarum Strains	Authors (Published Year)	Study Type (Single blind, Double blind, etc.)	Website	Country	Samples Size (Details : Size, Sex, Age, etc.)	Group Study Allocation (No. of groups, Size of each group, Ratio of Intervention : Control (I : C))	The Diseases of The Study	Duration of the Intervention
27	299v	K. KAZMIERCZAK-SIEDLECKA (2020)	A double-blind, randomized, and placebo controlled study	https://doi.org/10.26355/eurrev_202009_23059	Poland	35 cancer patients receiving home enteral nutrition were randomly assigned to intervention (n=21) and the placebo (n=14).	I : C = 21 : 14	Lp299v in cancer patients may reduce the gastrointestinal symptoms related to enteral nutrition and may improve laboratory parameters, predominantly the concentration of albumin	4 weeks intervention period.
28	MH-301	Kaige Zhang (2023)	A single-center, randomized, double-blind, and placebo-controlled clinical trial.	https://doi.org/10.1039/d3fo02936k	China	A total of 103 participants were randomly assigned to intervention (n=51) and the control (n=52).	I : C = 51 : 52	The administration of =MH-301 after endoscopic sclerotherapy can further increase the efficacy of the procedure and improve bowel movements.	4 weeks intervention period.
29	299v (DSM 9843)	Karolina Kaźmierczak-Siedlecka (2020)	A randomized, double-blind, and placebo-controlled trial	https://doi.org/10.1186/s12937-020-00598-w	Poland.	40 subjects were randomly allocated into 2 group, the intervention (n=20) and the placebo (n=20).	I : C = 20 : 20	The effect of 299v on prevention of weight loss of cancer patients receiving home enteral nutrition.	The 12 weeks intervention period
30	PC26	Katarina Meštrovic´ Popovicˇ (2022)	A double-blind, randomized, placebo-controlled clinical pilot study.	https://doi.org/10.3390/jcm11237008	Slovenia	Thirty children were enrolled and divided into two groups of intervention and the placebo.	I : C = 14 : 16	The probiotics might be helpful in alleviating Urinary Tract Infections (UTI) symptoms and in UTI prevention.	The 6 months intervention period
31	OLL2712	Keisuke Sakurai (2022)	A randomized, double-blind, placebo-controlled trial	https://doi.org/10.3390/nu14204300	Japan	81 participants were randomly assigned to intervention (n=41) and the placebo (n=40).	I : C = 41 : 40	OLL2712 ingestion has protective effects against memory function decline in older adults.	12 weeks intervention period. During October 2020 and March 2021 for recruitment and follow-up.

Item	Lactobacillus plantarum Strains	Authors (Published Year)	Study Type (Single blind, Double blind, etc.)	Website	Country	Samples Size (Details : Size, Sex, Age, etc.)	Group Study Allocation (No. of groups, Size of each group, Ratio of Intervention : Control (I : C))	The Diseases of The Study	Duration of the Intervention
32	APsulloc 331261 (GTB1(TM))	Kyoungmi Jung (2022)	A randomized, double-blind, and placebo-controlled trial	https://doi.org/10.3390/nu14102015	Korea	Twenty-seven participants were randomized for intervention or placebo.	I : C = 18 : 9	GTB1 enhanced intestinal discomfort symptoms, defecation consistency, quality of life, beneficial microbiota, and overall intestinal health.	The four weeks intervention and follow-up for two weeks. The study was conducted between November 2020 and January 2022
33	P-8	L.Y. Kwok (2014)	N.A.	https://doi.org/10.3920/bm2014.0063	China	A total of 33 subjects were recruited for the study.	All subjects were given a single daily oral dose of Lp-8 (6×1010 cfu) for 4 weeks.	This study evaluated the effect of consuming the Lp-8, on the faecal bacterial structure of the host. A beneficial shift in the faecal bacterial composition was detected.	The 4 weeks intervention period.
34	P-8	Lee-Ching Lew (2018)	A randomized, double-blind and placebo-controlled study	https://doi.org/10.1016/j.clnu.2018.09.010	Malaysia	103 subjects were randomly assigned to intervention (n=52) and the placebo (n=51).	I : C = 52 : 51	P8 is a feasible and natural intervention for the alleviation of selected stress, anxiety, memory and cognitive symptoms in stressed adults.	12 weeks intervention period.
35	P-8	Lifeng Wang M.Sc. (2013)	N.A.	https://doi.org/10.1016/j.nut.2013.11.018	China	Thirty-three recruited individuals for the study.	The subjects were given a single daily oral dose of Lp-8 (6 x10^10 colony forming units) for 4 wk.	Consumption of Lp-8 modulated fecal microbiota, SIgA, SCFAs, and TBAs in healthy individuals.	The experiment lasted for 8 weeks, the first 4 weeks consumes probiotics and next 4 weeks no probiotics consumes.

Item	Lactobacillus plantarum Strains	Authors (Published Year)	Study Type (Single blind, Double blind, etc.)	Website	Country	Samples Size (Details : Size, Sex, Age, etc.)	Group Study Allocation (No. of groups, Size of each group, Ratio of Intervention : Control (I : C))	The Diseases of The Study	Duration of the Intervention
36	CECT 7527, 7528 and 7529, mixture of 3 strains in the same proportion.	Mari C. Fuentes (2012).	A controlled, randomised, double-blind trial.	https://doi.org/10.1017/s000711451200373x	Spain	A total of 60 volunteers were equally randomly divided to intervention and placebo group (n=30).	I : C = 30 : 30	The supplementation of the diet with L. plantarum may contribute significantly to the reduction of serum cholesterol in hypercholesterolaemic patients.	12 weeks intervention period.
37	CECT 7527, 7528 and 7529, mixture of 3 strains in the same proportion.	Mari C. Fuentes (2016)	A double-blind, placebo-controlled, randomized trial,	https://DOI:10.3233/MNM-160065	Spain	A total of 60 subjects were randomly assigned to intervention (n=30) and the placebo (n=30)	I : C = 30 : 30	The L. plantarum combination reduced LDL-C and improved other lipid parameters, suggesting its potential for hypercholesterolemia treatment.	12 weeks intervention period and after 4 weeks follow-up period.
38	IMC 510	Maria Magdalena Coman (2022)	A randomized, placebo-controlled study	https://doi.org/10.1111/jam.15703	Italy	19 overweight/obese volunteers, 10 males and 9 females	The probiotic group of n=12 and Control group of n=7. (I : C = 12:7)	Lp. IMC 510 showed lowering effects on body weight and other measures of subjects with obese tendencies, suggesting its beneficial influence on metabolic disorders.	4 weeks run-in period followed by 12 weeks intervention period.
39	PS128	Martina Maria Mensi (2021)	The study was conducted without a randomization of participants.	https://doi.org/10.3390/nu13062036	Italy	A sample of 131 autistic children and adolescents (M:F = 122:19) were divided into intervention of PS128 or LP group (n=105) and intervention of other probiotics or OP group (n=26).	I (LP group) : C (OP group) = 105 : 26	Patients taking Lp-PS128 had greater improvements and fewer side effects than those taking other probiotics.	6 months intervention period.

Item	Lactobacillus plantarum Strains	Authors (Published Year)	Study Type (Single blind, Double blind, etc.)	Website	Country	Samples Size (Details : Size, Sex, Age, etc.)	Group Study Allocation (No. of groups, Size of each group, Ratio of Intervention : Control (I : C))	The Diseases of The Study	Duration of the Intervention
40	K50	Minji Sohn (2022)	A randomized, double-blind, and placebo-controlled trial	https://doi.org/10.3389%2Ffendo.2021.790046	South Korea	A total of 81 participants were randomly assigned to intervention (n=41) and the placebo (n= 40).	I : C = 41 : 40	Lp K-50 reduced the total cholesterol and triglyceride levels significantly.	12 weeks intervention period.
41	LMT1-48	Minji Sohn (2023)	A randomized, double-blind, placebo-controlled clinical trial,	https://doi.org/10.4093/dmj.2021.0370	Korea	The 100 volunteers were assigned randomly (1:1) to intervention or the placebo treatment group.	I : C = 50 : 50	The LMT1-48 decreased body weight, abdominal VFA, insulin resistance, and leptin levels suggesting its anti-obesogenic therapeutic potential.	12 weeks of treatment.
42	HAC01	Mi-Ra Oh (2021)	This randomized, double-blind, placebo-controlled clinical trial was conducted.	https://doi.org/10.3390/nu13072337	Korea	40 subjects were randomly assigned to receive a daily placebo (n = 20) or a dose of Lp HAC01 (n = 20) over eight weeks.	I: C = 20:20	HAC01 supplementation significantly improved HbA1c and 2h-PPG levels relative to placebo in prediabetic subjects.	It was conducted between July 2019 and November 2020. The intervention was conducted over an eight-week period.
43	299v	Mobin Malik (2018)	Eligible subjects were allocated in a non-randomized manner for the study.	https://doi.org/10.1161/circresaha.118.313565	USA	First, 20 men were recruited for the study who consumed Lp299v once daily for 6 weeks following by a 4-week washout period. Next, only 13 subjects continued for further study of oral liquid Vancomycin for 10 days.	N.A.	Lp299v improved vascular endothelial function and decreased systemic inflammation in men with coronary artery disease (CAD).	The 6 weeks intervention period, following 4 weeks washout. The study recruited subjects between 2013–2015.
44	299v	Moeen-Ul-Haq (2022)	The double-blind randomised control trial (RCT)	https://doi.org/10.47391/jpma.0758	Pakistan	The 120 subjects were randomly assigned to intervention (n=60) and the placebo (n=60).	I : C = 60 : 60	There was no significant improvement in the IBS symptoms among these treated with L. plantarum compared to those on placebo.	The study was conducted from July 20, 2014, to January 20, 2015.

Item	Lactobacillus plantarum Strains	Authors (Published Year)	Study Type (Single blind, Double blind, etc.)	Website	Country	Samples Size (Details : Size, Sex, Age, etc.)	Group Study Allocation (No. of groups, Size of each group, Ratio of Intervention : Control (I : C))	The Diseases of The Study	Duration of the Intervention
45	IS-10506	P.D. Kusumo (2019)	A randomised, double-blinded placebo-controlled trial	https://doi.org/10.3920/bm2017.0178	Indonesia	Total of 38 children were randomly assigned into 4 group of the placebo (n=11), probiotic (n=9), zinc (n=8) and probiotic and zinc group (n=10).	I(probiotic) : I(zinc) : I(probiotic and zinc) : C = 9 : 8 : 10 : 11	IS-10506 supplementation stimulates TGF-β1, which in turn increases the production of sIgA, in line with the significant correlation between TGF-β1/TNF-α and faecal sIgA.	90 days supplementation period.
46	LB931	Per Daniel Johannes Ronnqvist (2005)	A randomized, placebo-controlled, double-blind, multicenter study	https://doi.org/10.1080/00016340600578357	Sweden	A total of 191 subjects were randomly assigned to intervention (n=95) and the placebo (n=96).	I : C = 95 : 96	High numbers of LB931 may contribute to a low vaginal pH and seem to have a negative influence on Group B streptococci. LB931 could be transferred from the panty liners to both the vagina and the labial fold.	Specified microbes were counted and vaginal pH was measured once a month for five consecutive months.
47	MF1298	Per G. Farup (2012)	A randomized double-blind, placebo-controlled, crossover trial	https://doi.org/10.1155/2012/214102	Norway	Sixteen participants (11 women) were randomly assigned to intervention and the placebo.	N.A.	The intake of MF 1298 was a direct effect of the microbe on the gut wall and not caused by changes in the fecal microbiota.	A one-week run-in period followed by randomization and two three-week treatment periods separated by four weeks washout period.
48	299v (DSM 9843)	Philippe Ducrotté (2012)	A double blind, placebo-controlled, parallel-designed study	https://doi.org/10.3748/wjg.v18.i30.4012	France	The 214 subjects were randomly assigned to either intervention (n=108) or placebo (n=106) for 4 wk.	I : C = 108 : 106	The benefit of L. plantarum 299v in the management of IBS.	4 weeks intervention period.

Item	Lactobacillus plantarum Strains	Authors (Published Year)	Study Type (Single blind, Double blind, etc.)	Website	Country	Samples Size (Details : Size, Sex, Age, etc.)	Group Study Allocation (No. of groups, Size of each group, Ratio of Intervention : Control (I : C))	The Diseases of The Study	Duration of the Intervention
49	IS-10506	Pratiwi Dyah Kusumo (2019)	A Randomized Double-Blind Controlled Trial (RCT) was conducted	http://www.ncbi.nlm.nih.gov/pmc/articles/pmc7049320/	Indonesia	There were 37 women, with functional constipation symptoms were randomly assigned to intervention (n=16) and the placebo group (n=21).	I : C = 16 : 21	IS 10506 supplementation influenced all the Short Chain Fatty Acid (SCFA) parameter (acetate, pro-pionate and butyrate).	21 days intervention period.
50	CCFM8724	Qiuxiang Zhang (2023)	A randomized, double-blind, placebo-controlled trial.	https://doi.org/10.1080/07315724.2022.2043200	China	A Total of 58 children were recruited and randomly assigned to probiotic and placebo for 1:1.	I : C = 29 : 29	CCFM8724 significantly reduced the amounts of S. mutans and C. albicans in saliva of children with Early childhood caries (ECC).	The intervention and washout periods were 28 days and 14 days, respectively.
51	P17630	R. VLADAREANU (2018)	A randomized double-blind placebo-controlled study	https://doi.org/10.26355/eurrev_201801_14128	Romania	93 women were randomly assigned into intervention (55.9 % or n=52) and placebo group (44.1% or n=,41).	I : C = 52 : 41	The administration of P17630 improves vagina colonization of acid lactic bacteria and suggests the use of this oral product to successfully prevent episodes of vulvovaginal candidiasis.	15 days intake and 15 days washout. The entire duration of the study was 90 days.
52	IS-10506	Reza Gunadi Ranuh (2022)	A randomized, double-blind placebo-controlled trial	https://doi.org/10.37290/ijpp2641-7197.17:42-46	Indonesia	Twenty-one human immunodeficiency virus infected children were divided into placebo (n=11) and probiotic groups (n=10)	I : C = 10 : 11	IS-10506 increases the regulation of regulatory T cell in human immunodeficiency virus infection in children receiving first-line antiretroviral therapy treatment.	The 6 weeks intervention period. Observation on each subject was conducted over 6 weeks
53	JYLP-326	Ruizhe Zhu (2023)	This is a randomly allocated the subjects study.	https://doi.org/10.3389/fimmu.2023.1158137	China	Sixty anxious students were enrolled and randomly allocated to the placebo group (n=30) and the probiotic group (n=30).	I : C = 30 : 30	The intervention of JYLP-326 could be an effective strategy to alleviate anxiety, depression, and insomnia.	The 3 weeks intervention period.

Item	Lactobacillus plantarum Strains	Authors (Published Year)	Study Type (Single blind, Double blind, etc.)	Website	Country	Samples Size (Details : Size, Sex, Age, etc.)	Group Study Allocation (No. of groups, Size of each group, Ratio of Intervention : Control (I : C))	The Diseases of The Study	Duration of the Intervention
54	299v	S Sen (2002)	A double- blind, placebo-controlled, cross-over.	https://doi.org/10.1023/a:1020597001460	UK	Twelve patients were randomized to receive four weeks of intervention or a placebo group. At the end of the first phase, patients were to switch to the other preparation for a further four weeks.	N.A.	299V did not appear to alter colonic fermentation or improve symptoms in patients with the irritable bowel syndrome (IBS).	The 4 weeks intervention period for each phase.
55	I1001 (CECT7504)	S. Palacios (2016)	A clinical open-label, prospective study of two non-randomized parallel cohorts	https://doi.org/10.1007/s10096-016-2715-8	Spain	(Cohort 1) 33 sexually women prescribed a standard single-dose 500 mg vaginal tablet of clotrimazole followed by vaginal tablets with I1001. (Cohort 2) 22 women of similar characteristics but prescribed single-dose clotrimazole only.	I : C = 33 : 22	The therapy with vaginal tablets with I1001 could increase the effectiveness of single-dose 500 mg clotrimazole at preventing recurrence of vaginally on Vulvovaginal Candidiasis (VVC)	Treatment for two consecutive months.
56	MF1298	Solveig C Ligaarden (2010).	A randomised double blind, placebo-controlled, crossover trial	https://doi.org/10.1186/1471-230x-10-16	Norway	A total of 19 subjects were randomly selected assigned to intervention (n=10), and the placebo group (n= 9)	I : C = 10 : 9	L. plantarum MF1298 might be an unfavourable strain and this should stimulate basic research on the molecular basis of probiotic properties.	A one-week run-in period followed by randomisation and two three-week treatment periods separated by a four-week washout period.
57	PS128	Szu-Kai Fu (2021)	A double-blind, randomized, placebo-controlled, counterbalanced, crossover trial experiment.	https://doi.org/10.3390/nu13114023	Taiwan	The PS128 treatment for 4 weeks (LT, n = 8), or placebo for 4 weeks (PT, n = 8) in the control.	I : C = 8 : 8	PS128 supplementation was associated with an improvement in muscle damage, renal damage, and oxidative stress caused by HM through microbiota modulation and related metabolites	The period of nutritional supplementation was 4 weeks.

Item	Lactobacillus plantarum Strains	Authors (Published Year)	Study Type (Single blind, Double blind, etc.)	Website	Country	Samples Size (Details : Size, Sex, Age, etc.)	Group Study Allocation (No. of groups, Size of each group, Ratio of Intervention : Control (I : C))	The Diseases of The Study	Duration of the Intervention
58	P9	Teng Ma (2023)	A randomized, double-blind, placebo-controlled study	https://doi.org/10.1016/j.phrs.2023.106755	China	163 patients diagnosed with chronic constipation (CC) were randomly divided into probiotic group (n =78) and placebo group (n =85)	I : C = 78 : 85	The constipation relief effect of P9 intervention was accompanied by desirable changes in the fecal metagenome and metabolome.	Each patient was 56 days intervention period: 14 days before randomization; 28 days of treatment; and 14 days of follow-up. This study was conducted between November 2020 to May 2021.
59	GMNL6	Wan-Hua Tsai (2021)	N.A.	https://doi.org/10.7150/ijms.51545	Taiwan	15 females (25-50 years old) were recruited. The left face used base cream and the right face used base cream including heat-killed GMNL6.	I : C = 15 : 15	The people had better skin conditions after 1 month L. plantarum-GMNL6 treatment	2 months study was performed.
60	PS128	Wen-Ching Huang (2019)	Conducted the double-blind experimental design	https://doi.org/10.3390/nu11020353	Taiwan	In Study I, 18 subjects were randomly divided them into two groups: a placebo group (n = 9) and a L. plantarum group (n = 9). Study II also recruited 16 subjects (n = 8 for each groups).	For Study I, the ratio of I:C = 9 : 9. For study II the ratio of I: C = 8 : 8.	PS128 has beneficial effected on exercise performance maintenance, made possible via modulation of inflammation, oxidation, and the metabolism.	In study I, eight weeks of programmed training during the preparation period; the last four weeks included indicated supplementation. In Study II, the protocol was similar; however, [illegible]

Item	Lactobacillus plantarum Strains	Authors (Published Year)	Study Type (Single blind, Double blind, etc.)	Website	Country	Samples Size (Details : Size, Sex, Age, etc.)	Group Study Allocation (No. of groups, Size of each group, Ratio of Intervention : Control (I : C))	The Diseases of The Study	Duration of the Intervention
61	TWK10	Wen-Ching Huang (2019)	A double-blind placebo-controlled experiment.	https://doi.org/10.3390/nu11112836	Taiwan	54 participants (27 men and 27 women) allocated to the placebo, low dose and high dose group (n = 18 per group)	I(low) : I(high) : C = 18 : 18 : 18	The change in body composition shifted in the healthy direction for TWK10 groups, especially for the high dose group, which showed that body fat significantly decreased and muscle mass significantly increased.	6 weeks intervention period.
62	PS128	Wen-Ching Huang (2020)	A randomized, double-blind experimental design.	https://doi.org/10.3390/nu12082315	Taiwan	20 participants were randomly divided into two groups the placebo group (PG; n = 10) and an L. plantarum group (LG; n = 10), as a parallel-group study.	I : C = 10 : 10	PS128 supplementation was associated with an improvement on endurance running performance through microbiota modulation and related metabolites.	The 4 weeks intervention period.
63	P9	Wenjun Liu (2022)	A randomized, double-blind, and placebo-controlled trial	https://doi.org/10.1155/2022/4144321	China	200 eligible volunteers were randomly assigned to a probiotic group (n=100) or a placebo group (n=100).	I : C = 100 : 100	Study the efficiency and possible mechanism of action of probiotics for chronic constipation,	The 28 days intervention period.

Item	Lactobacillus plantarum Strains	Authors (Published Year)	Study Type (Single blind, Double blind, etc.)	Website	Country	Samples Size (Details : Size, Sex, Age, etc.)	Group Study Allocation (No. of groups, Size of each group, Ratio of Intervention : Control (I : C))	The Diseases of The Study	Duration of the Intervention
64	Q180	Ye Eun Park (2020)	A double-blind, randomized, placebo-controlled study	https://doi.org/10.3390/nu12010255	Korea	70 participants of both sexes were randomly allocated to intervention and the placebo group (n=35 each).	I : C = 35 : 35	LPQ180 might be developed as a functional ingredient to help maintain healthy postprandial lipid levels through modulating gut environment.	12 weeks intervention period after a two-week run-in period.
65	PS128	Yen-Wenn Liu (2019)	A randomized, double-blind, placebo-controlled study	https://doi.org/10.3390/nu11040820	Taiwan	The 80 subjects were randomly assigned to intervention (n=39) and the placebo (n=41).	I : C = 39 : 41	It seems that psychobiotic PS128 may be beneficial for children with autism spectrum disorder (ASD).	The 4 weeks intervention period.
66	CJLP133	Youngshin Han (2012)	A randomized, double-blind, placebo-controlled study	https://doi.org/10.1111/pai.12010	Korea	Total of 118 patients were randomly assigned to intervention (n=58) and the placebo group (n=60).	I : C = 58 : 60	The supplementation with CJLP133 is beneficial in the treatment of pediatric atopic dermatitis (AD).	16 weeks study period.
67	PS128	Yu-Ting Ho (2021)	A Randomized, Double-Blind, Placebo-Controlled Pilot Trial	https://doi.org/10.3390/nu13082820	Taiwan	Forty participants were randomly assigned to two groups, a PS128 group (n=21) and a placebo group (n=19).	I : C = 21 : 19	Daily consumption of PS128 as a dietary supplement may improve the depressive symptoms and sleep quality of insomniacs.	30 days intervention period.

Item	Lactobacillus plantarum Strains	Authors (Published Year)	Study Type (Single blind, Double blind, etc.)	Website	Country	Samples Size (Details : Size, Sex, Age, etc.)	Group Study Allocation (No. of groups, Size of each group, Ratio of Intervention : Control (I : C))	The Diseases of The Study	Duration of the Intervention
68	299v	K. Niedzielin (2001)	A controlled, double-blind, randomized study	http://dx.doi.org/10.1097/00042737-200110000-00004	Poland	Forty patients were randomized to receive either LP299V in liquid suspension (20 patients) or placebo (20 patients) over a period of 4 weeks	I : C = 20 : 20	LP299V seems to have a beneficial effect in patients with IBS	4 weeks intervention period.
69	DR7	Tatiana Altadill (2021)	A Post-Hoc Analysis of a Randomized, Placebo-Controlled Trial	https://doi.org/10.1096/fasebj.2021.35.S1.00121	Spain	Probiotic intervention (n=56) resulted in significantly fewer patient-days of URTI compared to placebo (n=53)	I : C = 56 : 53	L. plantarum DR7 significantly reduced patient-days with URTI by 20%, and patient-days with fever by 27%.	3-month intervention
